# Lobocrassins A–E: New Cembrane-Type Diterpenoids from the Soft Coral *Lobophytum crassum*

**DOI:** 10.3390/md9081319

**Published:** 2011-08-05

**Authors:** Chia-Ying Kao, Jui-Hsin Su, Mei-Chin Lu, Tsong-Long Hwang, Wei-Hsien Wang, Jih-Jung Chen, Jyh-Horng Sheu, Yueh-Hsiung Kuo, Ching-Feng Weng, Lee-Shing Fang, Zhi-Hong Wen, Ping-Jyun Sung

**Affiliations:** 1 Graduate Institute of Marine Biotechnology, National Dong Hwa University, Pingtung 944, Taiwan; E-Mails: chiaying1229@gmail.com (C.-Y.K.); x2219@nmmba.gov.tw (J.-H.S.); jinx6609@nmmba.gov.tw (M.-C.L.); 2 National Museum of Marine Biology and Aquarium, Pingtung 944, Taiwan; E-Mail: whw@nmmba.gov.tw (W.-H.W.); 3 Division of Marine Biotechnology, Asia-Pacific Ocean Research Center, National Sun Yat-sen University, Kaohsiung 804, Taiwan; E-Mails: sheu@mail.nsysu.edu.tw (J.-H.S.); wzh@mail.nsysu.edu.tw (Z.-H.W.); 4 Graduate Institute of Natural Products, Chang Gung University, Taoyuan 333, Taiwan; E-Mail: htl@mail.cgu.edu.tw; 5 Department of Marine Biotechnology and Resources, National Sun Yat-sen University, Kaohsiung 804, Taiwan; 6 Department of Pharmacy, Tajen University, Pingtung 907, Taiwan; E-Mail: jjchen@mail.tajen.edu.tw; 7 Tsuzuki Institute for Traditional Medicine, College of Pharmacy, China Medical University, Taichung 404, Taiwan; E-Mail: kuoyh@mail.cmu.edu.tw; 8 Department of Life Science and Institute of Biotechnology, National Dong Hwa University, Hualien 974, Taiwan; E-Mail: cfweng@mail.ndhu.edu.tw; 9 Department of Sport, Health, and Leisure, Cheng Shiu University, Kaohsiung 833, Taiwan; E-Mail: lsfang@csu.edu.tw

**Keywords:** lobocrassin, cembrane, *Lobophytum crassum*, cytotoxicity, superoxide anion, elastase

## Abstract

Five new cembrane-type diterpenoids, lobocrassins A–E (**1**–**5**), were isolated from the soft coral *Lobophytum crassum*. The structures of cembranes **1**–**5** were established by spectroscopic and chemical methods and by comparison of the spectral data with those of known cembrane analogues. Lobocrassin A (**1**) is the first cembranoid possessing an α-chloromethyl-α-hydroxy-γ-lactone functionality and is the first chlorinated cembranoid from soft corals belonging to the genus *Lobophytum*. Lobocrassins B (**2**) and C (**3**) were found to be the stereoisomers of the known cembranes, 14-deoxycrassin (**6**) and pseudoplexaurol (**7**), respectively. Lobocrassin B (**2**) exhibited modest cytotoxicity toward K562, CCRF-CEM, Molt4, and HepG2 tumor cells and displayed significant inhibitory effects on the generation of superoxide anion and the release of elastase by human neutrophils.

## 1. Introduction

**Figure 1.**
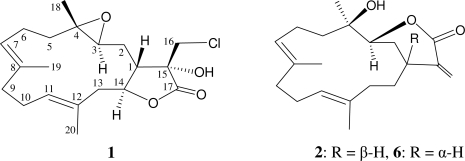

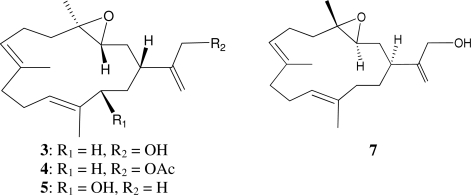
The structures of lobocrassins A–E (**1**–**5**), 14-deoxycrassin (**6**), and pseudoplexaurol (**7**).

Among the diterpenoids isolated from octocorals, the cembrane-type metabolites are the largest group of compounds [1], and the soft coral *Lobophytum crassum* (family Alcyoniidae) has been proven to be a rich source of cembrane-type compounds [2–13]. In our continuing research on novel substances from the octocorals distributed in the waters of Taiwan at the intersection of the Kuroshio current and the South China Sea surface current, the soft coral *L. crassum* was studied to determine the properties of its organic extract, which displayed cytotoxicity toward MCF-7 (human breast adenocarcinoma) and HeLa (human cervical carcinoma) cells (IC_50_ = 10.2 and 8.8 μg/mL, respectively). Five new cembrane derivatives, lobocrassins A–E (**1**–**5**) (Figure 1), were isolated. In this paper, we report the isolation, structure determination, and bioactivity of cembranes **1**–**5**.

## 2. Results and Discussion

Lobocrassin A (**1**) was isolated as a colorless oil, and the molecular formula for this compound was determined to be C_20_H_29_ClO_4_ (six units of unsaturation) using HRESIMS (C_20_H_29_ ^35^ClO_4_ + H, *m/z* 369.1830, calculated 369.1833). Comparison of the ^13^C NMR and DEPT data with the molecular formula indicated that there must be an exchangeable proton, which required the presence of a hydroxy group. This deduction was supported by a broad absorption in the IR spectrum at 3385 cm^−1^. The IR spectrum also showed a strong band at 1778 cm^−1^, consistent with the presence of a γ-lactone moiety. The ^13^C NMR data for **1** confirmed the presence of twenty carbon signals (Table 1), characterized by DEPT as three methyls, seven sp^3^ methylenes, two sp^2^ methines, three sp^3^ methines, three sp^2^ quaternary carbons, and two sp^3^ quaternary carbons. Based on the ^1^H and ^13^C NMR spectra (Table 1), **1** was determined to possess a γ-lactone (δ_C_ 173.4, C-17) and two trisubstituted olefins (δ_H_ 5.23, 1H, dd, *J* = 6.4, 6.4 Hz, H-11; 5.07, 1H, dd, *J* = 6.4, 6.4 Hz, H-7; δ_C_ 135.2, C-8; 130.2, CH-11; 130.1, C-12; 122.5, CH-7). The presence of a trisubstituted epoxide containing a methyl substituent was established from the signals of an oxygenated quaternary carbon (δ_C_ 64.0, C-4) and an oxymethine (δ_H_ 2.86, 1H, dd, *J* = 8.4, 4.4 Hz; δ_C_ 60.3, CH-3), and it was confirmed by the proton signal of a methyl singlet at δ_H_ 1.34 (3H, s, H_3_-18). Thus, from the reported data, the proposed skeleton of **1** was suggested to be a cembrane-type diterpenoid with three rings.

From the ^1^H–^1^H COSY spectrum of **1** (Table 1), it was possible to differentiate among the separate spin systems of H-3/H_2_-2/H-1/H-14/H_2_-13, H_2_-5/H_2_-6/H-7, and H_2_-9/H_2_-10/H-11. These data, together with the key HMBC correlations between protons and quaternary carbons of **1**, such as H_2_-2, H-5a, H-6a/C-4; H_2_-6, H_2_-9, H_2_-10/C-8; H_2_-10, H_2_-13, H-14/C-12; H-2a, H_2_-16, OH-15/C-15; and H_2_-16, OH-15/C-17, permitted the elucidation of the carbon skeleton. The vinyl methyls attached at C-8 and C-12 were confirmed by the HMBC correlations between H-7, H_2_-9/C-19; H_3_-19/C-7, C-8, C-9; and H-11/C-20; H_3_-20/C-11, C-12, C-13 and were further supported by the allylic couplings between H-7/H_3_-19 and H-11/H_3_-20. The C-3/4 epoxide group was confirmed by the HMBC correlations between H_2_-2, H_2_-5/C-3; H_2_-2, H-5a, H-6a/C-4; and H_3_-18/C-3, C-4, C-5. The presence of a hydroxy group at C-15 was deduced from the HMBC correlations between the hydroxy proton (δ_H_ 4.03, br s, OH-15) with C-1, C-15, C-16, and C-17.

The intensity of hydrogenated molecular (M + 2 + H)^+^ isotope peaks observed in the ESIMS and HRESIMS spectra [(M + H)^+^:(M + 2 + H)^+^ = 3:1] provided strong evidence for the presence of a chlorine atom in **1**. The methylene unit at δ_C_ 44.5 (CH_2_-16) was more shielded than expected for an oxygenated C-atom and was correlated to the methylene protons at δ_H_ 3.79 (H-16a) and 3.53 (H-16b) in the HMQC spectrum. These two protons showed a typical geminal coupling pattern with each other (*J* = 11.6 Hz), and these two proton signals were ^2^*J*-correlated with C-15 and ^3^*J*-correlated with C-1 and C-17 in the HMBC spectrum, demonstrating the attachment of a chlorine atom at C-16. Based on the above findings, the molecular framework of **1** was established unambiguously.

**Figure 2.**
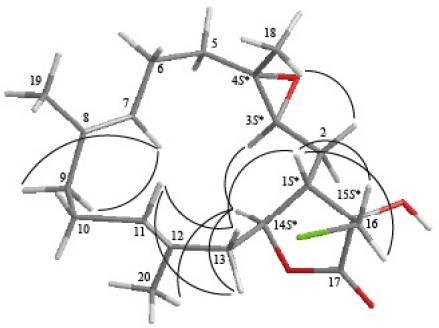
Computer-generated model for **1** using MM2 force field calculations and key NOESY correlations.

The relative configuration of **1** was elucidated from the interactions observed in a NOESY experiment. Most naturally occurring cembrane-type natural products from soft corals belonging to the order Alcyonacea have the H-1 in the β-orientation [14]. In the NOESY experiment for **1** (Figure 2), correlations observed between H-7 and H_2_-9 and H-11 and H_2_-13, as well as the lack of correlation between H-7/H_3_-19 and H-11/H_3_-20, reflected the *E* geometry of the double bonds at C-7/8 and C-11/12. Additionally, H-1 correlated with H-13b (δ_H_ 2.52), whereas H-14 showed responses to H-13a (δ_H_ 2.67), and the absence of correlation between H-1 and H-14 suggested a *trans*-fused γ-lactone in **1**. Moreover, it was found that H-14 showed interactions with H-3 and H_3_-20. Thus, assuming the α-orientation of H-14, H-3 should be positioned on the α face. In addition, H_3_-18 was found to interact with H-2a (δ_H_ 2.14), but not with H-3, revealing the *trans* geometry of the trisubstituted epoxide. H-1 correlated with H-16a/b, indicating that the C-16 methylene was situated on the β face in **1**. Based on the above findings, the structure of **1** was elucidated and the chiral centers for **1** were assigned as 1S*, 3*S* *, 4S*, 14S*, and 15S*.

In previous studies, chlorinated cembranoids have rarely been found [15–17]. To the best of our knowledge, lobocrassin A (**1**) is therefore the first cembranoid possessing an α-chloromethyl-α-hydroxy-γ-lactone functionality, and this compound is also the first chlorinated cembranoid from soft corals belonging to the genus *Lobophytum*.

Cembranoid **2** (lobocrassin B), obtained as a colorless oil, showed an (M + Na)^+^ signal at *m/z* 341.2091 in the HRESIMS, suggesting the molecular formula C_20_H_30_O_3_ (calcd C_20_H_30_O_3_ + Na, 341.2093), with six units of unsaturation. The IR absorptions of **2** at 3453 and 1721 cm^−1^ indicated the presence of hydroxy and δ-lactone functionalities. Through detailed analysis, cembranoid **2** had the same molecular formula as that of a well-known cembrane metabolite, 14-deoxycrassin (**6**), which was first isolated from the Caribbean gorgonian coral *Pseudoplexaura porosa* [18]. It was subsequently found that the spectral data of **2** were similar to those of **6**. However, by comparison of the optical rotation values and ^13^C NMR chemical shifts of the C-1 methine of **2** ( ${[\alpha ]}_{D}^{25}$ −40 (*c* 0.07, CHCl_3_); δ_C_ 35.5, CH-1) with that of **6** ( ${[\alpha ]}_{D}^{26}$ +29.6 (*c* 0.24, CHCl_3_); δ_C_ 33.23, CH-1), it was shown that the C-1 methine proton in **2** was β-oriented. Therefore, this compound should possess structure **2**. The structure of **2** was further confirmed by 2D NMR experiments (Table 2), and the chiral centers for this compound were assigned as 1R*, 3S*, and 4R*.

The NMR data of **3** (lobocrassin C) were in full agreement with those of a known cembrane analog, pseudoplexaurol (**7**), which was first isolated from the Caribbean gorgonian coral *Pseudoplexaura porosa* [18] and subsequently synthesized [19]. However, the optical rotation value of **3** ( ${[\alpha ]}_{D}^{24}$ +17 (*c* 0.37, CHCl_3_)) was substantially different from that of **7** ( ${[\alpha ]}_{D}^{26}$ −21.5 (*c* 3.4, CHCl_3_)), suggesting that **3** was an enantiomer of **7**. In the NOESY spectrum of **3**, H-3 showed a correlation with H-1, but not with H_3_-18, indicating that H-1 and H-3 were β-oriented and H_3_-18 was α-oriented in **3**. Thus, the chiral centers for **3** should be assigned as 1R*, 3R*, and 4R*.

Lobocrassin D (**4**) had a molecular formula of C_22_H_34_O_3_ as determined by HRESIMS at *m/z* 347.2580 (calcd for C_22_H_34_O_3_ + H, 347.2588). Detailed analysis of the spectral data showed that the data for **4** were similar to those of lobocrassin C (**3**). However, the signals corresponding to the 16-hydroxy group in **3** (δ_H_ 4.06, 2H, br s; δ_C_ 64.6, CH_2_-16) was replaced by those of an acetoxy group (δ_H_ 4.52, 1H, d, *J* = 20.8 Hz; 4.49, 1H, d, *J* = 20.8 Hz; δ_C_ 65.5, CH_2_-16; δ_H_ 2.08, 3H, s, acetate methyl; δ_C_ 170.6, acetate carbonyl; 21.0, acetate methyl) in **4**. Furthermore, acetylation of **3** gave a less polar product, which was found to be identical with natural product **4** and confirmed as cembranoid **4**.

Lobocrassin E (**5**) has the same molecular formula as that of **3**, C_20_H_30_O_2_, as determined by HRESIMS at *m/z* 327.2298 (calcd for C_20_H_30_O_2_ + Na, 327.2300) and with six units of unsaturation. These results indicated that compounds **3** and **5** were isomers. By comparison of the NMR data of **5** (Table 3) with those of **3**, the hydroxymethyl group in **3** (δ_H_ 4.06, 2H, br s; δ_C_ 64.6, CH_2_-16) was replaced by a vinyl methyl (δ_H_ 1.71, 3H, s; δ_C_ 18.8, CH_3_-16) in **5**, and the C-13 methylene in **3** (δ_H_ 2.11, 1H, m; 1.93, 1H, m; δ_C_ 35.0, CH_2_-13) was replaced by an oxymethine in **5** (δ_H_ 4.19, 1H, m; δ_C_ 76.6, CH-13). As mentioned for **1**, H-1 was suggested to be on the β face in **5**. In the NOESY experiment of **5**, H-3 exhibited correlations with H-1 and H-13 and no correlation was observed between H-3 and H_3_-18. From consideration of molecular models, H-3 was found to be reasonably close to H-1 and H-13 when H-3 was β-oriented and H-13 was placed on the α face. Based on the above findings, the relative configurations of the chiral centers for **5** were assigned as 1R*, 3R*, 4R*, and 13S*. In a previous study, a ketone analogue of cembranoid **5**, (1S*,3S*,4S*,7*E*,11*Z*)-3,4-epoxy-13-oxo-7,11,15-cembratriene, was isolated from an unidentified South Pacific soft coral [20]. Lobocrassin E (**5**) was subsequently proven to be an epimer of the alcohol derivative of (1S*,3S*,4S*, 7*E*,11*Z*)-3,4-epoxy-13-oxo-7,11,15-cembratriene.

The cytotoxicity of cembanes **1**–**4** toward K562 (human erythromyeloblastoid leukemia), CCRF-CEM (human T-cell acute lymphoblastic leukemia), Molt4 (human acute lymphoblastic leukemia), HepG2 (human hepatocellular liver carcinoma), and Huh 7 (human hepatocellular liver carcinoma) tumor cells were studied, and the results are shown in Table 4. The data show that lobocrassin B (**2**) exhibited modest cytotoxicity against K562, CCRF-CEM, Molt4, and HepG2 cells.

In addition, the *in vitro* anti-inflammatory effects of cembranes **1**–**5** were tested. Lobocrassin B (**2**) displayed significant inhibitory effects on the generation of superoxide anion and the release of elastase by human neutrophils (Table 5).

## 3. Experimental Section

### 3.1. General Experimental Procedures

Optical rotations were measured on a Jasco P-1010 digital polarimeter. Infrared spectra were recorded on a Varian Diglab FTS 1000 FT-IR spectrometer; peaks are reported in cm^−1^. The NMR spectra were recorded on Varian Mercury Plus 400 or Varian Inova 500 NMR spectrometers using the residual CHCl_3_ signal (δ_H_ 7.26 ppm) as an internal standard for ^1^H NMR and CDCl_3_ (δ_C_ 77.1 ppm) for ^13^C NMR. Coupling constants (*J*) are given in Hz. ^1^H and ^13^C NMR assignments were supported by ^1^H–^1^H COSY, HMQC, HMBC, and NOESY experiments. ESIMS were recorded on a Thermo Finnigan LCQ ion trap or a Bruker APEX II mass spectrometer. HRESIMS data were recorded on Thermo Fischer Scientific LTQ Orbitrap XL or a Bruker APEX II mass spectrometers. Column chromatography was performed on silica gel (230–400 mesh, Merck, Darmstadt, Germany). TLC was carried out on precoated Kieselgel 60 F_254_ (0.25 mm, Merck), and spots were visualized by spraying with 10% H_2_SO_4_ solution followed by heating. HPLC was performed using a system comprised of a Hitachi L-7100 pump, a Hitahci L-7455 photodiode array detector, and a Rheodyne injection port. A normal phase column (Hibar 250 × 10 mm, Merck, silica gel 60, 5 μm) was used for HPLC.

### 3.2. Animal Material

Specimens of the soft corals *L. crassum* were collected by hand using scuba equipment off the coast of northeast Taiwan at a depth of 10 m in August 2007 and stored in a freezer until extraction. A voucher specimen (NMMBA-TW-SC-2007-33) was deposited in the National Museum of Marine Biology and Aquarium, Taiwan.

### 3.3. Extraction and Isolation

The soft coral *L. crassum* (wet weight, 1.3 kg) was collected and freeze-dried. The material was minced and extracted with ethyl acetate (EtOAc). The EtOAc layer was separated on silica gel and eluted using *n*-hexane/EtOAc (stepwise from 100:1 to 0:100 *n*-hexane/EtOAc) to obtain 12 fractions. Fraction 8, eluted with *n*-hexane/EtOAc (1:1), was further separated by normal-phase HPLC (NP-HPLC) (*n*-hexane/EtOAc, 7:2) to afford **1** (1.9 mg). Compounds **2** (1.0 mg), **3** (7.3 mg), and **5** (1.2 mg) were obtained from fraction 6 by NP-HPLC (*n*-hexane/EtOAc, 4:1). Fraction 4, eluted with *n*-hexane/EtOAc (15:1–10:1), was separated on a silica gel column and further purified by NP-HPLC (*n*-hexane/EtOAc, 22:1) to yield **4** (1.7 mg).

Lobocrassin A (**1**): colorless oil; ${[\alpha ]}_{D}^{25}$ +28 (*c* 0.63, CHCl_3_); IR (neat) ν_max_ 3385, 1778 cm^−1^; ^1^H (CDCl_3_, 400 MHz) and ^13^C (CDCl_3_, 100 MHz) NMR data, see Table 1; ESIMS: *m/z* 369 (M + H)^+^, 371 (M + 2 + H)^+^; HRESIMS: *m/z* 369.1830 (calcd for C_20_H_29_ ^35^ClO_4_ + H, 369.1833).

Lobocrassin B (**2**): colorless oil; ${[\alpha ]}_{D}^{25}$ −40 (*c* 0.07, CHCl_3_); IR (neat) ν_max_ 3453, 1721 cm^−1^; ^1^H (CDCl_3_, 500 MHz) and ^13^C (CDCl_3_, 125 MHz) NMR data, see Table 2; ESIMS: *m/z* 341 (M + Na)^+^; HRESIMS: *m/z* 341.2091 (calcd for C_20_H_30_O_3_ + Na, 341.2093).

Lobocrassin C (**3**): colorless oil; ${\left[\alpha \right]}_{D}^{24}$ +17 (*c* 0.37, CHCl_3_); IR (neat) ν_max_ 3348 cm^−1^; ^1^H (CDCl_3_, 400 MHz) δ_H_ 5.09 (1H, dd, *J* = 6.4, 6.4 Hz, H-11), 5.08 (1H, d, *J* = 1.2 Hz, H-17a), 5.07 (1H, dd, *J* = 6.4, 6.4 Hz, H-7), 4.89 (1H, dd, *J* = 1.2, 0.8 Hz, H-17b), 4.06 (2H, br s, H_2_-16), 2.81 (1H, dd, *J* = 9.6, 3.6 Hz, H-3), 2.27 (1H, dddd, *J* = 8.8, 8.8, 6.0, 2.4 Hz, H-1), 2.19 (5H, m, H_2_-6, H-9a, and H_2_-10), 2.11 (1H, m, H-13a), 2.06 (1H, m, H-5a), 1.99 (1H, m, H-9b), 1.93 (1H, m, H-13b), 1.79 (1H, ddd, *J* = 14.4, 8.8, 3.6 Hz, H-2a), 1.73 (1H, m, H-14a), 1.64 (1H, m, H-14b), 1.61 (3H, s, H_3_-19), 1.59 (3H, s, H_3_-20), 1.50 (1H, ddd, *J* = 14.4, 9.6, 2.4 Hz, H-2b), 1.28 (1H, ddd, *J* = 11.6, 10.4, 3.6 Hz, H-5b), 1.24 (3H, s, H_3_-18); ^13^C (CDCl_3_, 100 MHz) δ_C_ 152.5 (C-15), 135.2 (C-8), 133.3 (C-12), 124.4 (CH-11), 123.7 (CH-7), 109.3 (CH_2_-17), 64.6 (CH_2_-16), 63.0 (CH-3), 60.7 (C-4), 39.5 (CH_2_-9), 38.3 (CH_2_-5), 37.2 (CH-1), 35.0 (CH_2_-13), 33.8 (CH_2_-2), 30.2 (CH_2_-14), 24.4 (CH_2_-10), 23.7 (CH_2_-6), 17.1 (CH_3_-20), 16.8 (CH_3_-18), 15.8 (CH_3_-19); ESIMS: *m/z* 327 (M + Na)^+^; HRESIMS: *m/z* 327.2299 (calcd for C_20_H_32_O_2_ + Na, 327.2300).

Lobocrassin D (**4**): colorless oil; ${\left[\alpha \right]}_{D}^{25}$ +71 (*c* 0.57, CHCl_3_); IR (neat) ν_max_ 1744 cm^−1^; ^1^H (CDCl_3_, 400 MHz) δ_H_ 5.09 (2H, dd, *J* = 7.2, 7.2 Hz, H-7 and H-11), 5.06 (1H, d, *J* = 1.6 Hz, H-17a), 4.94 (1H, s, H-17b), 4.52 (1H, d, *J* = 20.8 Hz, H-16a), 4.49 (1H, d, *J* = 20.8 Hz, H-16b), 2.82 (1H, dd, *J* = 10.0, 3.2 Hz, H-3), 2.29 (1H, m, H-1), 2.20 (1H, m, H-9a), 2.19 (2H, m, H_2_-10), 2.08 (2H, m, H_2_-6), 2.08 (3H, s, acetate methyl), 2.06 (1H, m, H-13a), 1.98 (1H, m, H-5a), 1.96 (1H, m, H-9b), 1.95 (1H, m, H-13b), 1.77 (1H, m, H-2a), 1.73 (1H, m, H-14a), 1.61 (3H, s, H_3_-19), 1.60 (1H, m, H-14b), 1.59 (3H, s, H_3_-20), 1.48 (1H, ddd, *J* = 14.0, 10.0, 2.4 Hz, H-2b), 1.29 (1H, m, H-5b), 1.25 (3H, s, H_3_-18); ^13^C (CDCl_3_, 100 MHz) δ_C_ 170.6 (acetate carbonyl), 135.2 (C-8), 147.3 (C-15), 133.1 (C-12), 124.3 (CH-11), 123.8 (CH-7), 112.6 (CH_2_-17), 65.5 (CH_2_-16), 62.9 (CH-3), 60.7 (C-4), 39.5 (CH_2_-9), 38.2 (CH_2_-5), 37.1 (CH-1), 34.7 (CH_2_-13), 34.1 (CH_2_-2), 30.4 (CH_2_-14), 24.4 (CH_2_-10), 23.7 (CH_2_-6), 21.0 (acetate methyl), 17.1 (CH_3_-20), 16.9 (CH_3_-18), 15.8 (CH_3_-19); ESIMS: *m/z* 347 (M + H)^+^; HRESIMS: *m/z* 347.2580 (calcd for C_22_H_34_O_3_ + H, 347.2588).

Lobocrassin E (**5**): colorless oil; ${\left[\alpha \right]}_{D}^{25}$ +47 (*c* 0.05, CHCl_3_); IR (neat) ν_max_ 3420 cm^−1^; ^1^H (CDCl_3_, 500 MHz) and ^13^C (CDCl_3_, 125 MHz) NMR data, see Table 3; ESIMS: *m/z* 327 (M + Na)^+^; HRESIMS: *m/z* 327.2298 (calcd for C_20_H_32_O_2_ + Na, 327.2300).

### 3.4. Acetylation of Lobocrassin C (**3**)

Lobocrassin C (**3**) (3.0 mg) was stirred with 2 mL of acetic anhydride in 2 mL of pyridine for 48 h at room temperature. After evaporation of excess reagent, the residue was separated by column chromatography on silica gel to give pure lobocrassin D (**4**) (*n*-hexane/EtOAc, 20:1, 3.3 mg, 97%); physical (*R_f_* and optical rotational values) and spectral (IR, ^1^H, and ^13^C NMR) data were in full agreement with those of natural product **4**.

### 3.5. Molecular Mechanics Calculations

Implementation of the MM2 force field [21] in CHEM3D PRO software from CambridgeSoft Corporation (Cambridge, MA, USA; ver 9.0, 2005) was used to calculate molecular models.

### 3.6. Cytotoxicity Testing

The cytotoxicity of compounds **1**–**4** was assayed with a modification of the MTT [3-(4,5-dimethylthiazol-2-yl)-2,5-diphenyltetrazolium bromide] colorimetric method. Cytotoxicity assays were carried out according to previously described procedures [22,23].

### 3.7. Superoxide Anion Generation and Elastase Release by Human Neutrophils

Human neutrophils were obtained by means of dextran sedimentation and Ficoll centrifugation. Measurements of superoxide anion generation and elastase release were carried out according to previously described procedures [24,25]. Briefly, superoxide anion production was assayed by monitoring the superoxide dismutase-inhibitable reduction of ferricytochrome *c*. Elastase release experiments were performed using MeO-Suc-Ala-Ala-Pro-Valp-nitroanilide as the elastase substrate.

## 4. Conclusions

In previous studies, a series of cembrane-type diterpenoids of potential medical interest were isolated from octocorals belonging to the genus *Lobophytum*. All corals, including reef-building corals and soft corals, are considered threatened species due to global climate change and habitat destruction. Therefore, the maintenance and culture of these interesting marine invertebrates as sources of new natural products of potential medical relevance is important. In our continuing search for novel substances from marine organisms originally collected from the Indo-Pacific Ocean, the hope is to identify extracts that exhibit interesting bioactivity. As an example, the bioactive cembranoid lobocrassin B (**2**) was isolated in this study. *L. crassum* was collected and transplanted back to tanks equipped with a flow-through sea water system. Advanced bioactivity testing for this compound will be carried out if sufficient material can be collected from culture-type species.

## Figures and Tables

**Table 1.** ^1^H and ^13^C NMR, ^1^H–^1^H COSY, and HMBC correlations for cembranoid **1**.

**C/H**	**^1^H^a^**	**^13^C^b^**		**^1^H–^1^H COSY**	**HMBC (H→C)**
1	2.76 ddd (10.0, 5.2, 4.0) ^c^	44.4	(CH) ^d^	H_2_-2, H-14	C-2, C-13, C-14, C-16
2a	2.14 ddd (15.6, 5.2, 4.4)	23.5	(CH_2_)	H-1, H-2b, H-3	C-1, C-3, C-4, C-14, C-15
b	1.68 ddd (15.6, 8.4, 4.0)			H-1, H-2a, H-3	C-1, C-3, C-4, C-14
3	2.86 dd (8.4, 4.4)	60.3	(CH)	H_2_-2	C-2
4		64.0	(C)		
5a	2.07 m	38.0	(CH_2_)	H-5b, H_2_-6	C-3, C-4, C-7
b	1.29 m			H-5a, H_2_-6	C-3
6a	2.24 m	23.4	(CH_2_)	H_2_-5, H-6b, H-7	C-4, C-7, C-8
b	2.09 m			H_2_-5, H-6a, H-7	C-7, C-8
7	5.07 dd (6.4, 6.4)	122.5	(CH)	H_2_-6, H_3_-19	C-6, C-9, C-19
8		135.2	(C)		
9a	2.26 m	38.8	(CH_2_)	H-9b, H_2_-10	C-7, C-8, C-10, C-11, C-19
b	2.04 m			H-9a, H_2_-10	C-7, C-8, C-10, C-11, C-19
10a	2.32 m	24.8	(CH_2_)	H_2_-9, H-10b, H-11	C-8, C-9, C-11, C-12
b	2.21 m			H_2_-9, H-10a, H-11	C-8, C-9, C-11, C-12
11	5.23 dd (6.4, 6.4)	130.2	(CH)	H_2_-10, H_3_-20	C-9, C-10, C-20
12		130.1	(C)		
13a	2.67 br d (14.4)	43.0	(CH_2_)	H-13b, H-14	C-1, C-11, C-12, C-14
b	2.52 dd (14.4, 7.2)			H-13a, H-14	C-1, C-11, C-12, C-14
14	4.66 ddd (10.0, 7.2, 2.8)	80.0	(CH)	H-1, H_2_-13	C-12
15		77.2	(C)		
16a	3.79 d (11.6)	44.5	(CH_2_)	H-16b	C-1, C-15, C-17
b	3.53 d (11.6)			H-16a	C-1, C-15, C-17
17		173.4	(C)		
18	1.34 s	17.0	(CH_3_)		C-3, C-4, C-5
19	1.60 s	15.7	(CH_3_)	H-7	C-7, C-8, C-9
20	1.74 s	17.4	(CH_3_)	H-11	C-11, C-12, C-13
OH-15	4.03 br s				C-1, C-15, C-16, C-17

a Spectra were measured at 400 MHz in CDCl_3_ at 25 °C;

b Spectra were measured at 100 MHz in CDCl_3_ at 25 °C;

c *J* values (in hertz) are in parentheses;

d Multiplicity was deduced by DEPT and HMQC experiments and indicated by the usual symbols.

**Table 2.** ^1^H and ^13^C NMR data, ^1^H–^1^H COSY, and HMBC correlations for cembranoid **2**.

**C/H**	**^1^H^a^**	**^13^C^b^**		**^1^H–^1^H COSY**	**HMBC (H→C)**
1	2.70 m	35.5	(CH) ^d^	H_2_-2, H-14	C-15, C-16, C-17
2	1.98 m	25.2	(CH_2_)	H-1, H-3	C-1, C-3, C-4, C-14, C-15
3	4.29 dd (8.0, 5.5) ^c^	79.9	(CH)	H_2_-2	C-1, C-4, C-5, C-18
4		74.6	(C)		
5a	1.87 m	37.4	(CH_2_)	H-5b, H_2_-6	C-3, C-4, C-6, C-7, C-18
b	1.68 ddd (14.5, 9.5, 4.5)			H-5a, H_2_-6	C-3, C-4, C-6, C-7, C-18
6a	2.22 m	22.4	(CH_2_)	H_2_-5, H-6b, H-7	C-4, C-5, C-7, C-8
b	2.16 m			H_2_-5, H-6a, H-7	C-4, C-5, C-7, C-8
7	5.21 dd (7.0, 7.0)	125.6	(CH)	H_2_-6, H_3_-19	C-5, C-6, C-9, C-19
8		135.7	(C)		
9	2.14 m	38.8	(CH_2_)	H_2_-10	C-7, C-8, C-10
10a	2.22 m	24.2	(CH_2_)	H_2_-9, H-10b, H-11	C-8, C-11, C-12
b	2.15 m			H_2_-9, H-10a, H-11	C-8, C-9, C-11, C-12
11	5.01 dd (6.5, 6.5)	124.7	(CH)	H_2_-10	C-10, C-13, C-20
12		135.3	(C)		
13a	2.21 m	36.1	(CH_2_)	H-13b, H_2_-14	C-14
b	2.02 m			H-13a, H_2_-14	C-1, C-11, C-12, C-14, C-20
14a	1.38 m	31.5	(CH_2_)	H-1, H_2_-13, H-14b	C-1, C-2, C-12
b	1.90 m			H-1, H_2_-13, H-14a	C-12
15		140.2	(C)		
16		166.5	(C)		
17a	6.34 s	125.7	(CH_2_)	H-17b	C-1, C-15, C-16
b	5.55 s			H-17a	C-1, C-16
18	1.27 s	24.2	(CH_3_)		C-3, C-4, C-5
19	1.56 s	15.3	(CH_3_)	H-7	C-7, C-8, C-9
20	1.61 s	15.6	(CH_3_)		C-11, C-12, C-13
OH-4	1.89 s				C-3, C-4, C-5, C-18

a Spectra were measured at 500 MHz in CDCl_3_ at 25 °C;

b Spectra were measured at 125 MHz in CDCl_3_ at 25 °C;

c *J* values (in hertz) are in parentheses;

d Multiplicity was deduced by DEPT and HMQC experiments and indicated by the usual symbols.

**Table 3.** ^1^H and ^13^C NMR data, ^1^H–^1^H COSY, and HMBC correlations for cembranoid **5**.

**C/H**	**^1^H^a^**	**^13^C^b^**		**^1^H–^1^H COSY**	**HMBC (H→C)**
1	2.05 m	39.3	(CH) ^d^	H_2_-2, H-14	n.o. ^e^
2a	1.89 ddd (14.5, 5.0, 4.0) ^c^	33.9	(CH_2_)	H-1, H-2b, H-3	C-1, C-3, C-4, C-14, C-15
b	1.46 ddd (14.5, 10.5, 3.5)			H-1, H-2a, H-3	C-1, C-3, C-4, C-14, C-15
3	2.85 dd (10.5, 4.0)	62.8	(CH)	H_2_-2	C-2, C-5
4		61.0	(C)		
5a	2.03 m	38.0	(CH_2_)	H-5b, H_2_-6	C-3, C-4, C-6, C-7
b	1.35 m			H-5a, H_2_-6	C-6, C-7
6a	1.99 m	23.1	(CH_2_)	H_2_-5, H-6b, H-7	C-4, C-7
b	2.17 m			H_2_-5, H-6a, H-7	C-7
7	5.11 dd (6.5, 6.5)	125.2	(CH)	H_2_-6, H_3_-19	C-6, C-9, C-19
8		134.6	(C)		
9	2.25 m	39.6	(CH_2_)	H_2_-10	C-8, C-11
10a	2.23 m	24.4	(CH_2_)	H_2_-9, H-10b, H-11	C-9, C-12
b	2.21 m			H_2_-9, H-10a, H-11	C-9, C-12
11	5.39 dd (7.0, 7.0)	128.7	(CH)	H_2_-10, H_3_-20	C-10, C-13, C-20
12		136.1	(C)		
13	4.19 m	76.6	(CH)	H_2_-14	n.o.
14	1.72 m	40.3	(CH_2_)	H-1, H-13	C-1, C-2, C-12, C-13, C-15
15		150.1	(C)		
16	1.71 s	18.8	(CH_3_)	H_2_-17	C-1, C-15, C-17
17a	4.68 s	109.8	(CH_2_)	H_3_-16, H-17b	C-1, C-16
b	4.65 s			H_3_-16, H-17a	C-1, C-16
18	1.20 s	17.6	(CH_3_)		C-3, C-4, C-5
19	1.62 s	15.3	(CH_3_)	H-7	C-7, C-8, C-9
20	1.62 s	10.5	(CH_3_)	H-11	C-11, C-12, C-13

a Spectra were measured at 500 MHz in CDCl_3_ at 25 °C;

b Spectra were measured at 125 MHz in CDCl_3_ at 25 °C;

c *J* values (in hertz) are in parentheses;

d Multiplicity was deduced by DEPT and HMQC experiments and indicated by the usual symbols;

e n.o. = not observed.

**Table 4.** Cytotoxicity of cembranes **1**–**4**.

**Compounds**	**Cell lines IC_50_(μg/mL)**				
	**K562**	**CCRF-CEM**	**Molt4**	**HepG2**	**Huh 7**
**1**	15.39	5.33	11.86	32.16	26.13
**2**	2.97	0.48	0.34	3.44	8.17
**3**	>40	11.55	9.51	>40	39.77
**4**	24.00	10.53	10.99	34.91	>40
Doxorubicin ^a^	0.24	0.05	0.07	0.71	0.46

a Doxorubicin was used as a reference compound. The results are expressed as mean ± S.D.

**Table 5.** Inhibitory effects of cembranes **1**–**5** on the generation of superoxide anion and the release of elastase by human neutrophils in response to formyl-Met-Leu-Phe/cytochalasin B (FMLP/CB).

**Compounds**	**Superoxide anion**	**Elastase release**
	**IC_50_(μg/mL) or (Inh %)^a^**	**IC_50_(μg/mL) or (Inh %)^a^**
**1**	(2.8 ± 1.9)	(0.9 ± 2.5)
**2**	4.8 ± 0.7	4.9 ± 0.4
**3**	(1.4 ± 2.4)	(9.6 ± 9.4)
**4**	(−1.9 ± 7.3)	(11.0 ± 3.9)
**5**	(−1.2 ± 1.5)	(−4.4 ± 9.5)
DPI ^b^	0.8 ± 0.2	
Elastatinal ^b^		30.8 ± 5.7

a Percentage of inhibition (Inh %) at a concentration 10 μg/mL;

b DPI (diphenylene indoniumn) and elastatinal were used as reference compounds. Results are expressed as mean ± S.E.M., and comparisons were made using Student’s *t*-test. A probability of ≤ 0.05 was considered significant.
